# Impact of chloride and strong ion difference on ICU and hospital mortality in a mixed intensive care population

**DOI:** 10.1186/s13613-016-0193-x

**Published:** 2016-09-17

**Authors:** Niels Van Regenmortel, Walter Verbrugghe, Tim Van den Wyngaert, Philippe G. Jorens

**Affiliations:** 1 Department of Intensive Care Medicine, Antwerp University Hospital, Wilrijkstraat 10, 2650 Edegem, Antwerp Belgium; 2 Department of Intensive Care Medicine, Ziekenhuis Netwerk Antwerpen Campus Stuivenberg, Lange Beeldekensstraat 267, 2060 Antwerp, Belgium; 3 Department of Nuclear Medicine, Antwerp University Hospital, Wilrijkstraat 10, 2650 Edegem, Antwerp Belgium; 4 Faculty of Medicine and Health Sciences, University of Antwerp, Universiteitsplein 1, 2610 Antwerp, Belgium

**Keywords:** Chloride, Hyperchloremia, Strong ion difference, Balanced solutions, Saline, NaCl 0.9 %, Mortality

## Abstract

**Background:**

Abnormal chloride levels are commonly observed in critically ill patients, but their clinical relevance remains a matter of debate. We examined the association between abnormal chloremia and ICU and hospital mortality. To further refine findings and integrate them into the ongoing discussion on the detrimental effects of chloride-rich solutions, the impact of strong ion difference (SID) on the same end points was assessed.

**Methods:**

Retrospective cohort study in an academic tertiary intensive care unit on 8830 adult patients who stayed at least 24 h in the ICU was carried out. Patients admitted after elective cardiac surgery were treated as a separate subgroup (*n* = 2350). Analyses were performed using multivariable logistic regression. All statistical models were extensively adjusted for confounders, including comorbidity, admission diagnosis, other electrolytes and acid–base parameters.

**Results:**

Severe hyperchloremia (>110 mmol/L), but not low (SID) was significantly associated with increased mortality in the ICU (odds ratio vs. normochloremia 1.81; 95 % CI 1.32–2.50; *p* < 0.001) and the hospital (odds ratio 1.49; 95 % CI 1.14–1.96; *p* = 0.003). Hyperchloremia and low (SID) were encountered in the majority of patients admitted after cardiac surgery (in 86.9 and 47.2 %, respectively), but were not negatively associated with mortality.

**Conclusions:**

In the ICU, hyperchloremia at admission was associated with negative outcome. On the other hand, decreased strong ion difference did not have an impact on mortality, precluding a simple extrapolation of these findings to the ongoing discussion on the detrimental effects of chloride-rich solutions. This notion is fueled by the finding that hyperchloremia after cardiac surgery, frequently encountered and probably fluid-induced, did not seem to be deleterious.

**Electronic supplementary material:**

The online version of this article (doi:10.1186/s13613-016-0193-x) contains supplementary material, which is available to authorized users.

## Background

Chloride is the electrolyte with the highest concentration in human plasma after sodium, yet its clinical importance is a subject of debate. Despite the high prevalence of abnormal chloride levels in the intensive care unit (ICU), research on the possible detrimental effects is scarce. Hyperchloremia has been shown to be negatively associated with mortality or kidney failure in certain subgroups of patients [1–3], although it is difficult to prove that it is an independent factor contributing to outcome rather than a mere marker of specific conditions or severity of illness [4, 5]. The fact that also hypochloremia has been linked to adverse outcomes in postoperative patients in a number of small-scale studies may point in this direction [6, 7]. Ignoring other electrolytes and acid–base parameters by considering chloride in isolation could be part of the problem.

Much of the interest is fueled by the fact that hyperchloremia can be induced by the intravenous (IV) administration of the widely used and economically attractive NaCl 0.9 % that contains chloride in a non-physiologically high amount [8–11]. There is a possibility to discern hyperchloremia caused by intravenous fluid use from many other causes by considering the relationship between sodium and chloride rather than treating the latter as a separate entity. The physicochemical approach introduced by the late Peter Stewart argues that the difference between strong cations and anions, by definition fully dissociated in human plasma, is one of the three independent determinants of pH [12]. This entity, termed strong ion difference or (SID), is reduced when hyperchloremia exists without an equal increase in sodium, e.g., after the intravenous administration of NaCl 0.9 %. Knowledge of the clinical impact of (SID) could contribute to the complex debate on the use of balanced solutions whose purpose is to prevent hyperchloremia and/or metabolic acidosis caused by a reduction in (SID). At this moment, despite being a well-known and independent cause of metabolic acidosis, the relevance of a decreased (SID) is doubted [13, 14].

The goal of our study was to analyze the clinical impact of abnormal chloremia and (SID) on the first day of admission in patients who stayed on a tertiary ICU for at least 24 h while taking other influential parameters into consideration. We assessed the link with mortality both on the ICU and in the hospital. Since this type of research could play a role in the ongoing debate on intravenous fluid therapy, we purposefully chose to include all patients regardless of the reason for their admission instead of preselecting subgroups of patients or diseases. The main aim of the study was to test the hypothesis that hyperchloremia, but also low (SID) are associated with negative outcomes.

## Methods

### Study design, study population and exclusion criteria

We conducted a large-scale retrospective cohort study in the 45-bed tertiary ICU at the Antwerp University Hospital, Belgium. The study was reviewed and approved by the hospital’s institutional review board (Number 14/9/88). Since all data were fully de-identified, the necessity of obtaining informed consent was waived. We collected data on every adult patient (over 16 years of age) staying on our ICU for at least 24 h between October 2007 and December 2014. Patients referred from another ICU and those receiving renal replacement therapy on their first day of admission were excluded. 10,165 patients were found eligible for the study (Additional file 1: Figure S1). We decided to divide the study population into two subgroups, one general ICU population, excluding those that underwent elective cardiac surgery, and a second including only the latter, as hyperchloremia after this type of surgery is common and probably fluid-induced.

### Data collection

All data were retrieved from the patient data management system (Metavision, iMDsoft, Düsseldorf, Germany). Demographic data included age, gender, Simplified Acute Physiology Score as marker of disease severity (SAPS-3) [15], admission type (medical, elective surgery, emergency surgery including trauma), an extensive comorbidity profile and the reason for admission, divided into 8 medical and 8 surgical categories (Additional file 2: Table S2). We also collected data on renal function by assessing the RIFLE score based on creatinine level [16]. Because this score can be troubled by methodological difficulties, e.g., the incomplete availability of baseline creatinine, we also calculated the RIFLE score based on urine production using an automated search for the 6- and 12-h time frame during which the smallest amount of urine was produced on the first day of admission [16]. Both scores were used separately in the models due to the much higher sensitivity of the urine-based score.

As regards laboratory data, we used only data from each patient’s first day of admission for statistical analysis. Since we were interested in chloride level, at least one measurement had to be taken during the first day. If multiple measurements were available, the highest value was used. Other variables included plasma biochemistry data (sodium, potassium, albumin, phosphate, calcium, magnesium) and arterial blood gas analysis (pH, pCO_2_, lactate). We only included laboratory data that had been taken within a certain time from the referenced chloride value: 1 h for sodium, potassium and blood gas parameters, and 24 h for albumin, phosphate, calcium and magnesium, although in the majority of cases all measurements were performed on the same sample.

To calculate the physicochemical acid–base parameters, we used generally accepted formulas and defined apparent SID as follows: SIDa = [Na^+^] + [K^+^] + [Ca^++^] + [Mg^++^] − [Cl^−^] (all values by convention in mEq/L) [12]. Since lactate is fully ionized in the pH range of 6–8, it also acts as a strong anion. We decided, however, to exclude lactate from the calculation of SIDa because we wished to specifically investigate the impact of the electrolytes. The inclusion of lactate could have led to ambiguous conclusions considering its well-established role as a marker of poor clinical outcome [17]. Effective SID was defined as the electrical charge attributed to the routinely measured weak anions and calculated as follows: SIDe = bicarbonate + albumin charge + phosphate charge (all values in mEq/L), where bicarbonate = 0.0301*pCO_2_*10^pH−6.1^, albumin charge = [albumin] in g/L*(pH*0.123–0.631) and phosphate charge = [phosphate] in mmol/L*(pH*0.309–0.469) [18, 19]. Strong ion gap (SIG), the sum of routinely unmeasured anions, was defined as SIG = SIDa − lactate − SIDe.

Arterial blood gases were analyzed on a point-of-care Rapidlab 1265 (Siemens Laboratory Diagnostics, Beersel, Belgium). Biochemistry analyses were performed using direct potentiometry (Vitros 950, Johnson & Johnson, Ortho Clinical Diagnostics, Beerse, Belgium) until April 2010, after which the central laboratory changed to indirect potentiometry (Dimension Vista 1500, Siemens Laboratory Diagnostics, Beersel, Belgium). To match samples taken before and after this transition, Passing-Bablok regression equations were applied to the measurements taken before April 2010. To exclude residual confounding [20, 21] due to this transition, we also added the potentiometry type as a covariate in the statistical models and double-checked all conclusions using analyses with point-of-care data that were not influenced by a change in measurement technique (Additional file 3: Table S3, Additional file 4: Table S4).

### Statistical analysis and end points

Statistical analyses were performed using STATA 14 (StataCorp LP, Texas, USA). End points were mortality in the ICU within 30 days of admission and hospital mortality. In the latter case, if a patient was admitted more than once, we kept only the last admission for analysis. Means were reported ± standard deviations (SD) and confidence intervals (CI) at the 95 % level. Patients missing any of the covariates were excluded from analysis (Additional file 1: Figure S1).

Before constructing each of the statistical models, we performed a visual inspection of the predictor–response relationship between the covariates of interest and the outcome variable using locally weighted scatter plot smoothing (lowess) [22]. If the shape of the curve suggested a nonlinear relationship and thus a detrimental effect on the end point for both low and high values, covariates were categorized. We decided to divide chloride levels into four categories based on the normal range for chloride in our institution: normochloremia (98–107 mmol/L), hypochloremia (<98 mmol/L), moderate hyperchloremia (107–110 mmol/L) and severe hyperchloremia (>110 mmol/L). We divided hyperchloremia into two categories because we observed that the mean chloride level in our population (107.3 ± 5.5 mmol/L) was at the high end of our normal range for chloride. In addition, a chloride level of >110 mmol/L is accepted in the literature as clinically relevant [1, 2]. Because a commonly accepted range of (SID) is lacking in the literature and SIDa was normally distributed in our population, we opted for cutoff points based on the range of one standard deviation around the mean value (42.9 ± 3.6): “low SIDa” < 41.1 mEq/L, “intermediate SIDa” 41.1–44.7 mEq/L and “high SIDa” > 44.7 mEq/L. We avoided the term “normal SIDa” as a decreased (SID) can be the result of an adequate metabolic compensation process.

Since the outcome variables were binary, we used multivariable logistic regression. We first constructed multivariable models with chloride and SIDa alone, to separate osmolality effects (where both sodium and chloride change in the same direction) from chloride load/acid–base effects and then tested covariates that are known to be associated with the outcome parameter. We only kept covariates that considerably improved model fit, as assessed by the −2 log likelihood and formal tests of equality of the area under the receiver operating characteristic curves (ROC). Covariates that are known to potentially influence the covariates of interest (e.g., heart failure treated with diuretics, renal failure, albumin level, chronic obstructive pulmonary disease and other chronic changes in pCO_2_ can affect chloride level and SIDa) were forced into the models regardless of their statistical impact. For each variable, the odds ratio (OR) was calculated with its 95 % CI. To ensure a correct model specification, we assessed the quadratic form of the linear predictor. All models passed the Hosmer–Lemeshow goodness-of-fit test. Because of the close relationship between some variables, variance inflation factors were used to identify collinear independent variables and reported with every model. Statistical significance was set at a *p* value of less than 0.05 (two-sided) for all tests.

## Results

**Fig. 1:**
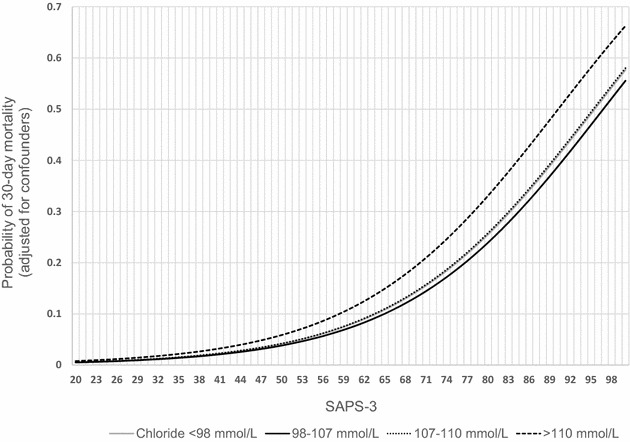
Probability of 30-day mortality per chloride category based on the logistic regression model in Table 2. Simulation using a lactate level of 2 mEq/L, an admission diagnosis associated with a high risk of death (e.g., sepsis; Additional file 2: Table S2), no comorbidities and a normal sodium level

Demographic data, clinical outcomes and biochemical values of the study population in the different admission types are summarized in Table 1. Mean chloride level was 107.3 ± 5.5 mmol/L and mean SID 42.9 ± 3.6 mEq/L. Table 2 provides the patients’ characteristics of the general ICU population according to chloride category. Remarkably, in an unadjusted analysis, patients already suffering from acute kidney injury during their first day of admission did not seem to experience more hyperchloremia than those without. The results of the regression models in the general ICU population (*n* = 6480) are presented in Table 3. There was a significant association between severe, but not moderate hyperchloremia and 30-day ICU mortality, with an 81 % increase in the odds of death versus normochloremia (*p* < 0.001). We visualized this model by plotting the relationship between the probability of death and SAPS-3 score for different categories of chloremia (Fig. 1). This effect was independent of hypernatremia, that was encountered notably less frequently than hyperchloremia (8.5 vs. 41.1 %). There was no significant association between low SIDa and mortality. Analyses with hospital mortality as the outcome parameter showed similar results (odds ratio of severe hyperchloremia versus normochloremia 1.46; 95 % CI 1.14–1.96; *p* = 0.003), although also hypochloremia and high SIDa showed a negative association. We also constructed a model in patients with hyperchloremia (>107 mmol/L), treating chloride level as a continuous variable, resulting in an OR of death of 1.06 per 1 mmol/L rise in chloride (95 % CI 1.01–1.11; *p* = 0.02). Finally, the possibility exists that not every fluid-related dyschloremia is reflected by assessing only the highest chloride level of the first day of admission, as this could neglect an additional resuscitation effort after the assessment. Therefore, we also performed an additional sensitivity analysis by constructing a statistical model using the highest chloride level and lowest SIDa of the first 2 days of admission. The results are presented in Additional file 5: Table S5 and confirm the main analysis.

**Table 1:** Clinical characteristics, outcome data and biochemistry values on day 1 of admission

	Entire cohort *n* = 10,165	Admission type			
		Medical *n* = 3505 (34.5 %)	Elective surgery		Emergency surgery and trauma *n* = 2109 (20.7 %)
			Cardiac	Other	
			*n* = 2357 (23.2 %)	*n* = 2194 (21.6 %)	
Demographic data					
Female (%)	3861 (38.0 %)	1369 (39.1 %)	754 (32.0 %)	914 (41.7 %)	824 (39.1 %)
Age (mean in years ± SD)	62.7 ± 15.3	63.0 ± 15.0	68.7 ± 10.7	60.1 ± 14.4	58.1 ± 17.0
SAPS-3 (mean ± SD)	49.5 ± 16.2	61.3 ± 14.5	40.5 ± 8.9	36.8 ± 10.9	53.1 ± 14.1
Comorbidities [*n* (%)]					
Chronic kidney disease	1317 (12.9 %)	600 (17.1 %)	297 (12.6 %)	187 (8.5 %)	233 (11.1 %)
Heart failure	2931 (28.8 %)	963 (27.19 %)	1392 (59.1 %)	217 (9.9 %)	369 (17.5 %)
COPD	1120 (11.0 %)	490 (14.0 %)	228 (9.7 %)	243 (11.1 %)	159 (7.5 %)
Diabetes mellitus	1743 (17.2 %)	671 (19.1 %)	489 (20.8 %)	290 (13.2 %)	293 (13.9 %)
Liver cirrhosis	348 (3.4 %)	202 (5.8 %)	10 (0.4 %)	63 (2.9 %)	73 (3.5 %)
Malignancy	440 (4.3 %)	96 (2.7 %)	11 (0.5 %)	283 (12.9 %)	50 (2.37 %)
Outcome [*n* (%)]					
30-day ICU mortality	929 (9.1 %)	622 (17.8 %)	54 (2.3 %)	44 (2.0 %)	209 (9.9 %)
Hospital mortality^a^	1661 (19.7 %)	1061 (37.6 %)	99 (4.5 %)	173 (10.0 %)	327 (19.1 %)
Covariates of interest on day of admission (mean ± SD)					
Chloride (mmol/L)	107.3 ± 5.5	104.6 ± 6.0	111.3 ± 3.4	107.6 ± 3.9	107.1 ± 5.3
SIDa (mEq/L)	42.9 ± 3.6	44.3 ± 4.0	41.2 ± 3.0	42.4 ± 2.9	43.1 ± 3.4
Biochemistry and physicochemical covariates on day of admission (mean ± SD)					
Sodium (mmol/L)	140.1 ± 4.9	138.8 ± 5.4	142.3 ± 3.7	139.9 ± 3.7	140.1 ± 5.2
Lactate (mmol/L)	2.4 ± 2.1	2.4 ± 2.5	2.8 ± 1.6	1.7 ± 1.1	2.5 ± 2.4
Albumin (g/L)	27.2 ± 7.0	28.5 ± 6.9	23.0 ± 5.0	28.5 ± 6.1	28.2 ± 8.0
SIDa incl. lactate (mEq/L)	40.6 ± 3.9	42.0 ± 4.3	38.4 ± 2.9	40.7 ± 3.0	40.7 ± 3.6
SIDe (mEq/L)	33.7 ± 4.9	34.8 ± 5.8	30.6 ± 3.3	34.7 ± 3.5	34.0 ± 4.6
SIG (mEq/L)	6.9 ± 3.5	7.2 ± 3.8	7.8 ± 2.9	5.9 ± 3.0	6.6 ± 3.7

*SAPS* Simplified Acute Physiology Score, *COPD* chronic obstructive pulmonary disease, *SIDa* apparent strong ion difference, *SIDe* effective strong ion difference, *SIG* strong ion gap

^a^If more than one ICU admission, only data on the last admission were used

**Table 2:** Patients’ characteristics according to chloride category

General ICU population (excl. cardiac surgery)	Hypochloremia	Normochloremia	Moderate hyperchloremia	Severe hyperchloremia
*n* = 6480	*n* = 434	*n* = 3382	*n* = 1456	*n* = 1206
Patient characteristics				
Age (mean ± SD)	63.0 ± 14.9	62.0 ± 15.7	60.1 ± 15.8	59.3 ± 15.9
SAPS-3 (mean ± SD)	59.2 ± 14.6	51.8 ± 17.1	48.2 ± 17.1	52.8 ± 17.7
Mechanical ventilation on day 2	25 (5.8 %)	149 (4.4 %)	83 (5.7 %)	87 (7.2 %)
Comorbidities				
COPD	113 (26.0 %)	427 (12.6 %)	140 (9.6 %)	74 (6.1 %)
Heart failure	140 (32.3 %)	699 (20.7 %)	193 (13.3 %)	150 (12.4 %)
Chronic kidney disease	90 (20.7 %)	422 (12.5 %)	170 (11.7 %)	144 (11.9 %)
Outcome				
30-day mortality	434 (16.4 %)	3382 (10.2 %)	1456 (8.5 %)	1208 (14.6 %)
Hospital mortality (*n* = 5184^a^)	329 (43.8 %)	2707 (24.3 %)	1188 (18.4 %)	960 (29 %)
Acute kidney injury [*n* (%)]				
Creatinine-based RIFLE score				
None (81.0 %)	300 (5.7 %)	2747 (52.3 %)	1237 (23.6 %)	967 (18.4 %)
Risk (9.3 %)	57 (9.4 %)	296 (48.9 %)	132 (21.8 %)	120 (19.8 %)
Injury (5.7 %)	39 (10.6 %)	193 (52.6 %)	53 (14.4 %)	82 (22.3 %)
Failure (4.0 %)	38 (14.8 %)	146 (56.8 %)	34 (13.2 %)	39 (15.2 %)
Urine-based RIFLE score				
None (14.4 %)	70 (7.5 %)	479 (51.34 %)	199 (21.3 %)	185 (19.8 %)
Risk (48.7 %)	195 (6.2 %)	1624 (51.5 %)	715 (22.7 %)	618 (19.6 %)
Injury (35.3 %)	144 (6.3 %)	1217 (53.2 %)	530 (23.2 %)	398 (17.4 %)
Failure (1.6 %)	25 (23.6 %)	62 (58.5 %)	12 (11.3 %)	7 (6.6 %)
Biochemistry and physicochemical covariates on day of admission				
pCO_2_				
Low (<35 mmHg)	96 (22.1 %)	773 (22.9 %)	359 (24.7 %)	372 (30.8 %)
Normal	186 (42.9 %)	1850 (54.7 %)	849 (58.3 %)	664 (55.0 %)
High (>45 mmHg)	152 (35.0 %)	759 (22.4 %)	248 (17.0 %)	172 (14.2 %)
Sodium (mmol/L)	132.3 ± 5.6	137.9 ± 3.2	141.1 ± 2.9	144.6 ± 4.5
SIDa (excl. lactate) (mEq/L)	47.7 ± 4.6	43.9 ± 3.1	42.3 ± 2.7	41.1 ± 3.5
Lactate (mmol/L)	3.1 ± 3.1	2.8 ± 2.6	2.7 ± 2.3	3.3 ± 2.8
Strong ion gap (SIG) (mEq/L)	6.9 ± 4.7	6.6 ± 3.3	6.4 ± 3.3	6.8 ± 3.8

*COPD* chronic obstructive pulmonary disease, *SIDa* apparent strong ion difference (excl. lactate)

^a^If more than one ICU admission, only data on the last admission were used

**Table 3:** Logistic regression models in the total ICU population excluding patients admitted after elective cardiac surgery

	Outcome: 30-day mortality^a^ (*n* = 6480)				Outcome: hospital mortality^b^ (*n* = 5184)			
	*n*	% outcome (%)	Odds ratio (95 % CI) versus normochloremia	*p* value	*n*	% outcome (%)	Odds ratio (95 % CI) versus normochloremia	*p* value
Chloride category								
Normochloremia	3382	10.2			2707	24.3		
Hypochloremia	434	16.4	1.02 (0.71–1.48)	0.91	329	43.8	1.55 (1.13–2.13)	0.007
Moderate hyperchloremia	1456	8.5	1.16 (0.88–1.51)	0.30	1188	18.4	0.96 (0.77–1.20)	0.72
Severe hyperchloremia	1208	14.6	1.81 (1.32–2.50)	<0.001	960	29.0	1.49 (1.14–1.96)	0.003

	*n*	% outcome (%)	Odds ratio (95 % CI) versus intermediate SIDa	*p* value	*n*	% outcome (%)	Odds ratio (95 % CI) versus intermediate SIDa	*p* value
SIDa category								
Intermediate SIDa	2982	9.6			2382	20.5		
Low SIDa	1598	9.4	0.89 (0.68–1.16)	0.40	1260	21.5	0.94 (0.70–1.15)	0.60
High SIDa	1900	14.6	1.05 (0.83–1.32)	0.71	1542	34.9	1.35 (1.11–1.65)	0.002
			Area under ROC 86.7 %				Area under ROC 83.5 %	
			Maximal VIF 2.26				Maximal VIF 2.39	
			Tolerance 0.68				Tolerance 0.68	

*SIDa* apparent strong ion difference (excl. lactate), *ROC* receiver under operating characteristics curve, *VIF* variance inflation factor

^a^Confounders adjusted for in the model: sodium (high vs. normal*, low vs. normal*), SAPS-3, admission reason, RIFLEcrea*, RIFLEurine, lactate, heart failure*, COPD*, pCO2* (low, normal, high), albumin, SIG, potentiometry type* (*p > 0.05)

^b^Confounders adjusted for in the model: sodium (high vs. normal*, low vs. normal*), SAPS-3, admission reason, RIFLEcrea, RIFLEurine, lactate, heart failure, COPD, pCO2* (low, normal, high), albumin, SIG*, potentiometry type (*p > 0.05)

The subgroup of patients that underwent elective cardiac surgery consisted of 2350 patients. Of note, the majority of patients experienced some form of hyperchloremia (*n* = 2042, 86.9 %) and chloride level was significantly higher than in the general study population (111.3 ± 3.4 vs. 106.1 ± 5.5 mmol/L; *p* < 0.001), while SIDa was lower (41.2 ± 3.0 vs. 43.4 ± 3.6 mmol/L; *p* < 0.001) (Table 1). Table 4 presents the results of the regression models. Neither hyperchloremia nor SIDa was significantly associated with 30-day mortality. With regard to hospital mortality, moderate (OR 0.43; *p* = 0.01) and severe hyperchloremia (OR 0.37; *p* = 0.004) were significantly associated with a decreased odds of death.

**Table 4:** Logistic regression models on the subgroup admitted after elective cardiac surgery

	Outcome: 30-day mortality^a^ (*n* = 2350)				Outcome: hospital mortality^b^ (*n* = 2156)			
	*n*	% outcome (%)	Odds ratio (95 % CI) versus normochloremia	*p* value	*n*	% outcome (%)	Odds ratio (95 % CI) versus normochloremia	*p* value
Chloride category								
Normochloremia	287	5.6			246	13.4		
Hypochloremia	21	23.8	3.49 (0.62–19.62)	0.16	15	40.0	1.45 (0.30–7.18)	0.64
Moderate hyperchloremia	596	3.0	0.63 (0.26–1.54)	0.31	550	5.1	0.43 (0.22–0.83)	0.01
Severe hyperchloremia	1446	2.7	0.57 (0.22–1.44)	0.23	1345	4.5	0.37 (0.18–0.73)	0.004

	*n*	% outcome (%)	Odds ratio (95 % CI) versus mean SID	*p* value	*n*	% outcome (%)	Odds ratio (95 % CI) versus mean SID	*p* value
SIDa category								
Intermediate SIDa	941	2.6			873	5.2		
Low SIDa	1110	2.0	1.46 (0.68–3.11)	0.34	1015	4.1	1.13 (0.63–2.03)	0.68
High SIDa	229	10.7	0.85 (0.39–1.83)	0.67	268	14.9	0.71 (0.38–1.31)	0.27
			Area under ROC 88.3 %				Area under ROC 83.9 %	
			Maximal VIF 3.53				Maximal VIF 3.58	
			Tolerance 0.66				Tolerance 0.66	

*SIDa* apparent strong ion difference (excl. lactate), *ROC* Receiver under Operating Characteristics Curve, *VIF* variance inflation factor

^a^Confounders adjusted for in the model: sodium (high vs. normal, low vs. normal*), SAPS-3, RIFLEcrea, lactate, heart failure*, COPD*, pCO2* (low, normal, high), albumin*, SIG, potentiometry type (*p > 0.05). RIFLEurine omitted because of collinearity

^b^Confounders adjusted for in the model: sodium (high vs. normal, low vs. normal*), SAPS-3, RIFLEcrea, lactate, heart failure*, COPD, pCO2* (low, normal, high), albumin*, SIG, potentiometry type (*p > 0.05). RIFLEurine omitted because of collinearity

## Discussion

We found a significant and consistent association between severe hyperchloremia and mortality in a large ICU population, confirming on a broader scale previous findings after non-cardiac surgery and sepsis [1, 2]. The inclusion of every patient staying at least 24 h in the ICU and thus the widest possible range of diagnoses and patient profiles extends the scope of the conclusions to include critically ill patients in general. As far as we know, this is the first study in a broad ICU population including the largest number of patients ever analyzed in this regard. The challenging question whether hyperchloremia has a causal role or is merely a consequence of certain conditions remains an issue that cannot be fully elucidated using our study design. The most obvious example of this chicken and egg situation can be found in kidney injury. While hyperchloremia could be a consequence of renal failure, it was also found to induce vasoconstriction of the afferent arteriole leading to the renal glomerulus and to reduce renal blood flow velocity and cortical perfusion [23, 24]. Our extensive dataset allowed for a precise and granular profiling of the included patients and their illness, and we also adjusted all statistical models for sodium level and kidney injury at admission, using two separate calculations of the RIFLE score (creatinine and diuresis). Also, the lack of a clearly increased prevalence of hyperchloremia in patients with acute kidney injury (Table 2) could be a step toward the answer to this question.

It could be tempting to add these findings to the growing evidence of the deleterious effect of chloride-rich solutions. However, a unilateral view on hyperchloremia, with its broad causal range, could lead to premature conclusions. We addressed this issue twofold. First, we assessed SIDa in conjunction with chloremia as it is unaltered by the change of sodium and chloride in the same direction and thus a more selective indicator of hyperchloremia induced by intravenous fluid use. SIDa is a difficult parameter to handle, as it can itself be the result of a physiological or compensatory process [25–27]. We therefore constructed statistical models that included relevant parameters (SIG, lactate, albumin, pCO_2_) and comorbidities. Second, we performed all analyses on a large subgroup of patients admitted after elective cardiac surgery of which we could assume most of the postoperative electrolyte disturbances were caused by intraoperative fluid manipulations. None of our statistical models showed a significant relationship between low SIDa and outcome. Additionally, in the cardiac surgery subgroup, hyperchloremia was not harmful and was even associated with a significant reduction of hospital mortality, although it is possible that hypoalbuminemia/hypernatremia-induced alkalosis in this subgroup mitigates hyperchloremia-induced metabolic acidosis. We believe this finding should urge clinicians to adopt a cautious approach in drawing conclusions in the debate on chloride-rich solutions based on findings considering only hyperchloremia. In different experiments, detrimental renal effects could be observed after a fast-administered, highly dosed amount of chloride-rich fluids [24, 28, 29]. Our data, reflecting real-life use and thus a broad range of fluid strategies for different clinical reasons, do not disprove the existence of this phenomenon, as we lacked a detailed fluid profile in our dataset. Also, our approach principally took fluid-induced metabolic acidosis into account, whereas the total amount of administered chloride could be an equally relevant culprit, as was shown in certain studies [30, 31], although one could expect a major chloride load to be reflected in chloride level.

Apart from the retrospective design, the possibility of still unmeasured confounding and the rather arbitrary cutoff for low SIDa, we regard our inability to decouple chloride level from pre-admission fluid therapy as the most important limitation of our study. It is plausible that a patient who develops hyperchloremia in the ICU after large volume resuscitation in the operating theater or emergency room will have a higher chance of mortality than a patient that is normochloremic without the need for a comparable resuscitation effort. Hyperchloremia could be a mere reflection of the amount of resuscitation fluids, itself being a marker of severity of shock and/or fluid overload and thus mortality. This limitation applies in the same way to the other recently published studies on the detrimental effects of hyperchloremia. In a retrospective cohort study, hyperchloremia at 72 h of ICU stay in septic patients was found to be associated with hospital mortality [2]. The lack of data on intravenous fluid administration during ICU stay could reduce the conclusion to the message that the amount of these fluids, rather than their chloride content, was associated with mortality. Likewise, in the abovementioned cohort study in postoperative patients, even careful propensity matching could probably not resolve the question whether the amount or the chloride content of intraoperative fluid was responsible for the observed mortality increase [1]. Even research on the effect of chloride load itself is unable to fully decouple chloride level from the administered fluid volume [30–32].

## Conclusions

In a general intensive care population, after careful controlling for confounders, hyperchloremia was significantly associated with increased 30-day ICU and hospital mortality. A low strong ion difference on the other hand, was not found to be linked with negative outcome, precluding a simplistic extrapolation of our and other findings on hyperchloremia to the ongoing discussion on the detrimental effects of chloride-rich solutions.

## Additional files

10.1186/s13613-016-0193-x Cohort derivation, exclusion criteria and missing values. ICU = intensive care unit.
10.1186/s13613-016-0193-x Admission type and admission reason categories.
10.1186/s13613-016-0193-x Logistic regression models in the general ICU population excluding patients admitted after elective cardiac surgery. Point-of-care data for chloride and sodium were used as covariates and for the calculation of SIDa.
10.1186/s13613-016-0193-x Logistic regression models on the subgroup admitted after elective cardiac surgery. Point-of-care data for chloride and sodium were used as covariates and for the calculation of SIDa.
10.1186/s13613-016-0193-x Logistic regression models in the total ICU population excluding patients admitted after elective cardiac surgery. The highest chloride and the lowest SIDa encountered during the first two days of admission were used for analysis.
