# Supplementary figures and images for: Impact of chloride and strong ion difference on ICU and hospital mortality in a mixed intensive care population
Source: Ann Intensive Care. 2016 Sep 17;6:91. doi: 10.1186/s13613-016-0193-x (PMC5026977; doi:10.1186/s13613-016-0193-x)

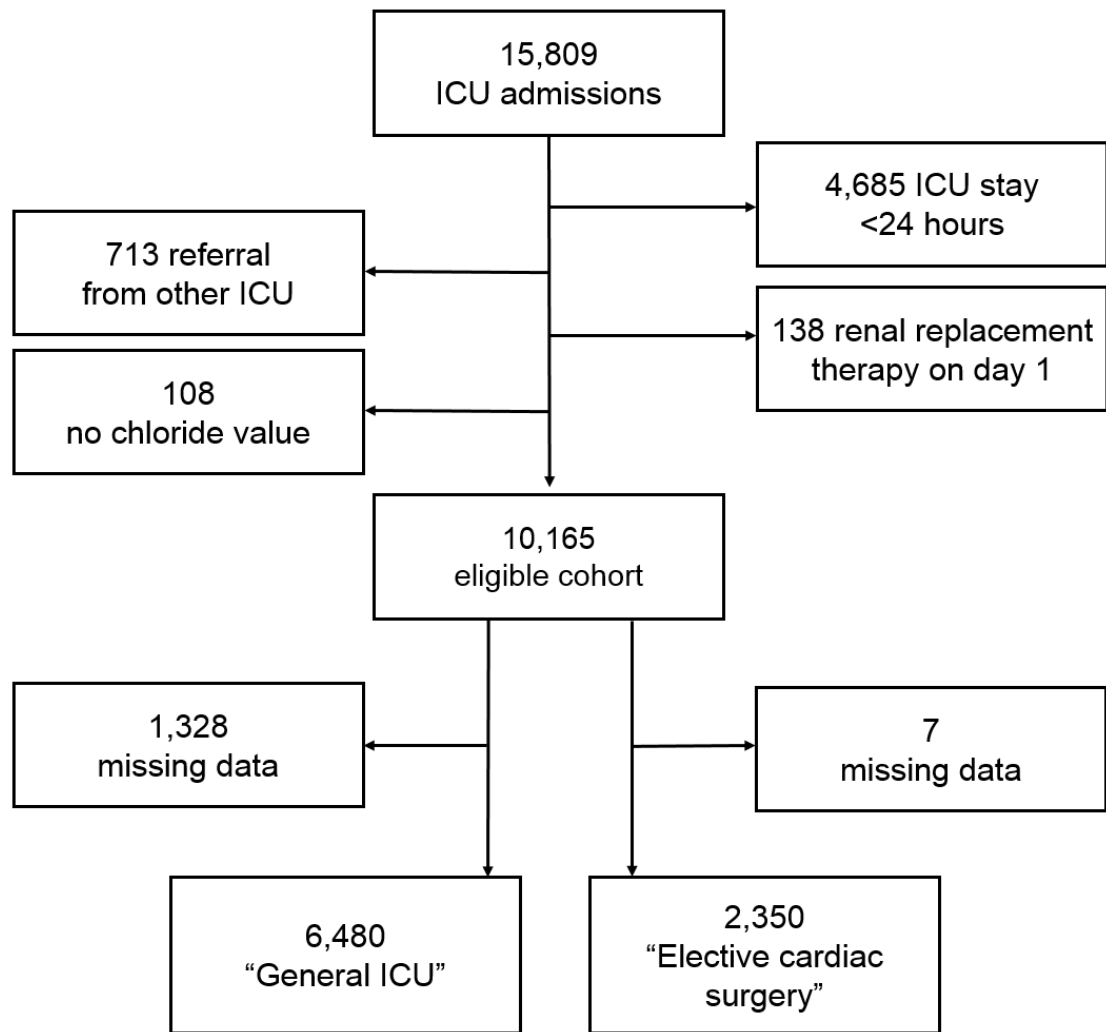

Figure S1: Cohort derivation, exclusion criteria and missing values. ICU = intensive care unit.

Supplement: Supplementary file 1 — 10.1186/s13613-016-0193-x Cohort derivation, exclusion criteria and missing values. ICU = intensive care unit. [file 13613_2016_193_MOESM1_ESM.pdf]
