# Supplementary material for: Impact of chloride and strong ion difference on ICU and hospital mortality in a mixed intensive care population
Source: Ann Intensive Care. 2016 Sep 17;6:91. doi: 10.1186/s13613-016-0193-x (PMC5026977; doi:10.1186/s13613-016-0193-x)
Supplement: Supplementary file 2 — 10.1186/s13613-016-0193-x Admission type and admission reason categories. [file 13613_2016_193_MOESM2_ESM.pdf]

Medical			Surgical				
				Elective		Emergency/Trauma	
Diagnostic category	n	(%)	Diagnostic category	n	%	n	%
Hemodynamic	1,210	35.5%	Abdominal	180	4.0%	249	11.8%
Hemorrhage	259	7.4%	Hepatic/Pancreatic	405	8.9%	91	4.3%
Respiratory	457	13%	**Cardiac**	**2,357**	**51.8%**	352	16.7%
Gastrointestinal	60	1.7%	Intracranial	496	10.9%	402	19.1%
Hepatic	121	3.5%	Thoracic	686	15.1%	69	3.3%
Sepsis	709	20.2%	Vascular	164	3.6%	325	15.4%
Neurological	384	11.0%	Trauma	0	0.0%	415	19.7%
Other	305	8.7%	Other	263	5.8%	206	9.8%
Total	3,505	100%	Total	4,551	100,0%	2,109	100,0%

Table S2: Admission type and admission reason categories
