# Supplementary material for: Impact of chloride and strong ion difference on ICU and hospital mortality in a mixed intensive care population
Source: Ann Intensive Care. 2016 Sep 17;6:91. doi: 10.1186/s13613-016-0193-x (PMC5026977; doi:10.1186/s13613-016-0193-x)
Supplement: Supplementary file 3 — 10.1186/s13613-016-0193-x Logistic regression models in the general ICU population excluding patients admitted after elective cardiac surgery. Point-of-care data for chloride and sodium were used as covariates and for the calculation of SIDa. [file 13613_2016_193_MOESM3_ESM.pdf]

| POC-DATA | Outcome: 30-day mortality[1] (n=6,465) | | | | Outcome: hospital mortality[2] (n=5,173) | | | |
|---|---|---|---|---|---|---|---|---|
| **Chloride category** | **n** | **% outcome** | **Odds ratio (95% CI) vs. normochloremia** | **p-value** | **n** | **% outcome** | **Odds ratio (95% CI) vs. normochloremia** | **p-value** |
| Normochloremia | 2.372 | 9.4% | | | 1.879 | 24.6% | | |
| Hypochloremia | 311 | 11.9% | 1.03 (0.69-1.60) | 0.88 | 242 | 37.6% | 1.24 (0.88-1.75) | 0.22 |
| Moderate hyperchloremia | 1.586 | 9.5% | 1.27 (0.97-1.66) | 0.08 | 1.297 | 20.5% | 1.09 (0.87-1.36) | 0.46 |
| Severe hyperchloremia | 2.196 | 13.7% | 1.58 (1.61-2.13) | 0.003 | 1.755 | 27.0% | 1.43 (1.10-1.85) | 0.007 |
| **SIDa category** | **n** | **% outcome** | **Odds ratio (95% CI) vs. intermediate SIDa** | **p-value** | **n** | **% outcome** | **Odds ratio (95% CI) vs. intermediate SIDa** | **p-value** |
| Intermediate SIDa | 2.747 | 10.3% | | | 2.210 | 22.1% | | |
| Low SIDa | 1.497 | 12.8% | 1.02 (0.79-1.32) | 0.87 | 1.164 | 26.5% | 0.92 (0.74-1.15) | 0.45 |
| High SIDa | 2.221 | 10.7% | 1.10 (0.85-1.41) | 0.47 | 1.799 | 27.7% | 1.36 (1.10-1.68) | 0.004 |
| **Sodium category** | **n** | **% outcome** | **Odds ratio (95% CI) vs. normal sodium** | **p-value** | **n** | **% outcome** | **Odds ratio (95% CI) vs. normal sodium** | **p-value** |
| Normal sodium | 4.312 | 9.9% | | | 3.497 | 22.6% | | |
| Hyponatremia | 1.815 | 11.7% | 1.28 (0.99-1.67) | 0.06 | 1.395 | 28.0% | 1.28 (1.03-1.58) | 0.02 |
| Hypernatremia | 338 | 21.9% | 1.21 (0.84-1.73) | 0.31 | 281 | 40.6% | 1.04 (0.75-1.44) | 0.81 |
| | | | Area under ROC 86.6% | | | | Area under ROC 83.4% | |
| | | | Maximal VIF 2.70 | | | | Maximal VIF 2.72 | |
| | | | Tolerance 0.67 | | | | Tolerance 0.67 | |

Table S3: Logistic regression models in the **general ICU population** excluding patients admitted after elective cardiac surgery. Point-of-care data for chloride and sodium were used as covariates and for the calculation of SIDa.

[1] Confounders adjusted for in the model: SAPS-3, admission reason, RIFLEcrea*, RIFLEurine, lactate, heart failure*, COPD*, pCO2* (low, normal, high), albumin, SIG, potentiometry type* (* = p > 0.05).

[2] Confounders adjusted for in the model: SAPS-3, admission reason, RIFLEcrea, RIFLEurine, lactate, heart failure, COPD, pCO2* (low, normal, high), albumin, SIG*, potentiometry type (* = p > 0.05).

SIDa = apparent strong ion difference (excl. lactate); ROC = Receiver under Operating Characteristics Curve; VIF = variance inflation factor
