# Supplementary material for: Impact of chloride and strong ion difference on ICU and hospital mortality in a mixed intensive care population
Source: Ann Intensive Care. 2016 Sep 17;6:91. doi: 10.1186/s13613-016-0193-x (PMC5026977; doi:10.1186/s13613-016-0193-x)
Supplement: Supplementary file 4 — 10.1186/s13613-016-0193-x Logistic regression models on the subgroup admitted after elective cardiac surgery. Point-of-care data for chloride and sodium were used as covariates and for the calculation of SIDa. [file 13613_2016_193_MOESM4_ESM.pdf]

POC-data	Outcome: 30-day mortality[1] (n=2,342)				Outcome: hospital mortality[2] (n=2,149)			
**Chloride category**	**n**	**% outcome**	**Odds ratio (95% CI) vs. normochloremia**	**p-value**	**n**	**% outcome**	**Odds ratio (95% CI) vs. normochloremia**	**p-value**
Normochloremia	110	6.4%			93	15.1%		
Hypochloremia	12	33.3%	10.85 (1.56-75.48)	0.016	7	71.4%	24.03 (3.00-192.12)	0.003
Moderate hyperchloremia	238	2.9%	0.86 (0.23-3.29)	0.83	218	6.0%	0.68 (0.26-1.82)	0.45
Severe hyperchloremia	1,982	3.0%	0.90 (0.26-3.14)	0.87	1,831	5.2%	0.52 (0.21-1.32)	0.17
**SIDa category**	**n**	**% outcome**	**Odds ratio (95% CI) vs. mean SID**	**p-value**	**n**	**% outcome**	**Odds ratio (95% CI) vs. mean SID**	**p-value**
Intermediate SIDa	1,005	3.4%			931	6.0%		
Low SIDa	1,138	2.1%	0.82 (0.43-1.56)	0.54	1,045	4.5%	0.98 (0.59-1.61)	0.92
High SIDa	199	10.1%	0.82 (0.36-1.89)	0.64	173	13.9%	0.67 (0.32-1.37)	0.27
**Sodium category**	**n**	**% outcome**	**Odds ratio (95% CI) vs. normal sodium**	**p-value**	**n**	**% outcome**	**Odds ratio (95% CI) vs. normal sodium**	**p-value**
Normal sodium	2,035	2.5%			1,869	4.7%		
Hyponatremia	198	5.6%	1.14 (0.42-3.10)	0.80	175	11.4%	1.22 (0.59-2.51)	0.60
Hypernatremia	109	14.7%	1.85 (0.77-4.49)	0.17	105	18.1%	1.94 (0.90-4.15)	0.09
			Area under ROC 87.9%				Area under ROC 83.3%	
			Maximal VIF 4.47				Maximal VIF 4.48	
			Tolerance 0.66				Tolerance 0.66	

Table S4: Logistic regression models on the subgroup admitted after **elective cardiac surgery**. Point-of-care data for chloride and sodium were used as covariates and for the calculation of SIDa.

[1] Confounders adjusted for in the model: SAPS-3, RIFLEcrea, lactate, heart failure*, COPD*, pCO2* (low, normal, high), albumin*, SIG, potentiometry type* (* = p > 0.05). RIFLEurine omitted because of collinearity.

[2] Confounders adjusted for in the model: SAPS-3, RIFLEcrea, lactate, heart failure, COPD, pCO2* (low, normal, high), albumin*, SIG, potentiometry type* (* = p > 0.05). RIFLEurine omitted because of collinearity.

SIDa = apparent strong ion difference (excl. lactate); ROC = Receiver under Operating Characteristics Curve; VIF = variance inflation factor
