# Supplementary material for: Impact of chloride and strong ion difference on ICU and hospital mortality in a mixed intensive care population
Source: Ann Intensive Care. 2016 Sep 17;6:91. doi: 10.1186/s13613-016-0193-x (PMC5026977; doi:10.1186/s13613-016-0193-x)
Supplement: Supplementary file 5 — 10.1186/s13613-016-0193-x Logistic regression models in the total ICU population excluding patients admitted after elective cardiac surgery. The highest chloride and the lowest SIDa encountered during the first two days of admission were used for analysis. [file 13613_2016_193_MOESM5_ESM.pdf]

Highest Chloride D1 & 2<br>Lowest SIDa D1 & 2	Outcome: 30-day mortality[1] (n=6,480)				Outcome: hospital mortality[2] (n=5,176)			
**Chloride category**	**n**	**% outcome**	**Odds ratio (95% CI) vs. normochloremia**	**p-value**	**n**	**% outcome**	**Odds ratio (95% CI) vs. normochloremia**	**p-value**
Normochloremia	3.087	9.9%			2.439	24.7%		
Hypochloremia	250	18.8%	1.52 (0.99-2.35)	0.06	190	50.5%	2.21 (1.51-3.24)	<0.001
Moderate hyperchloremia	1.570	8.7%	1.14 (0.87-1.48)	0.35	1.285	18.7%	0.91 (0.74-1.14)	0.42
Severe hyperchloremia	1.573	14.3%	1.67 (1.24-2.24)	0.001	1.262	28.1%	1.32 (1.03-1.68)	0.03
**SIDa category**	**n**	**% outcome**	**Odds ratio (95% CI) vs. intermediate SIDa**	**p-value**	**n**	**% outcome**	**Odds ratio (95% CI) vs. intermediate SIDa**	**p-value**
Intermediate SIDa	3.065	9.8%			2.459	21.2%		
Low SIDa	2.011	9.8%	0.97 (0.76-1.24)	0.81	1.587	21.7%	0.86 (0.80-1.20)	0.86
High SIDa	1.404	15.5%	1.07 (0.83-1.37)	0.60	1.130	38.0%	1.44 (1.17-1.76)	<0.001
**Sodium category**	**n**	**% outcome**	**Odds ratio (95% CI) vs. normal sodium**	**p-value**	**n**	**% outcome**	**Odds ratio (95% CI) vs. normal sodium**	**p-value**
Normal sodium	4.921	9.4%			3.963	22.5%		
Hyponatremia	1.009	13.0%	1.22 (0.92-1.61)	0.17	761	29.2%	0.89 (0.70-1.23)	0.32
Hypernatremia	550	21.8%	1.07 (0.75-1.48)	0.68	452	39.8%	1.07 (0.80-1.42)	0.67
			Area under ROC 86.7%				Area under ROC 83.6%	
			Maximal VIF 2.38				Maximal VIF 2.40	
			Tolerance 0.68				Tolerance 0.68	

Table S5: Logistic regression models in the **total ICU population** excluding patients admitted after elective cardiac surgery. The highest chloride and the lowest SIDa encountered during the **first two days of admission** were used for analysis.

[1] Confounders adjusted for in the model: SAPS-3, admission reason, RIFLEcrea*, RIFLEurine, lactate, heart failure*, COPD*, pCO2* (low, normal, high), albumin, SIG, potentiometry type* (* = p > 0.05).

[2] Confounders adjusted for in the model: SAPS-3, admission reason, RIFLEcrea, RIFLEurine, lactate, heart failure, COPD, pCO2* (low, normal, high), albumin, SIG*, potentiometry type (* = p > 0.05).

SIDa = apparent strong ion difference (excl. lactate); ROC = Receiver under Operating Characteristics Curve; VIF = variance inflation factor
